# CASP11 – An Evaluation of a Modular BCL::Fold-Based Protein Structure Prediction Pipeline

**DOI:** 10.1371/journal.pone.0152517

**Published:** 2016-04-05

**Authors:** Axel W. Fischer, Sten Heinze, Daniel K. Putnam, Bian Li, James C. Pino, Yan Xia, Carlos F. Lopez, Jens Meiler

**Affiliations:** 1 Department of Chemistry, Vanderbilt University, Nashville, TN, 37232, United States of America; 2 Center for Structural Biology, Vanderbilt University, Nashville, TN, 37232, United States of America; 3 Department of Biomedical Informatics, Vanderbilt University, Nashville, TN, 37232, United States of America; 4 Chemical and Physical Biology Graduate Program, Vanderbilt University, Nashville, TN, 37232, United States of America; 5 Department of Cancer Biology and Center for Quantitative Sciences, Vanderbilt University, Nashville, TN, 37232, United States of America; University of Michigan, UNITED STATES

## Abstract

*In silico* prediction of a protein’s tertiary structure remains an unsolved problem. The community-wide *Critical Assessment of Protein Structure Prediction* (*CASP*) experiment provides a double-blind study to evaluate improvements in protein structure prediction algorithms. We developed a protein structure prediction pipeline employing a three-stage approach, consisting of low-resolution topology search, high-resolution refinement, and molecular dynamics simulation to predict the tertiary structure of proteins from the primary structure alone or including distance restraints either from predicted residue-residue contacts, nuclear magnetic resonance (NMR) nuclear overhauser effect (NOE) experiments, or mass spectroscopy (MS) cross-linking (XL) data. The protein structure prediction pipeline was evaluated in the *CASP11* experiment on twenty regular protein targets as well as thirty-three ‘assisted’ protein targets, which also had distance restraints available. Although the low-resolution topology search module was able to sample models with a *global distance test total score* (GDT_TS) value greater than 30% for twelve out of twenty proteins, frequently it was not possible to select the most accurate models for refinement, resulting in a general decay of model quality over the course of the prediction pipeline. In this study, we provide a detailed overall analysis, study one target protein in more detail as it travels through the protein structure prediction pipeline, and evaluate the impact of limited experimental data.

## Introduction

*In silico* prediction of a protein’s tertiary structure from its sequence remains an unsolved problem. The vast size of the conformational space that needs to be sampled with limited CPU cycles requires simplifications in sampling and scoring, often in conjunction with a simplified representation of the protein. Consequently, the depth of the native energy minimum is reduced making it difficult to distinguish it from alternative energy minima [1–5]. The limited sampling density results in an intrinsic, minimal deviation of the conformations sampled and the lowest energy conformation that exists in each region of the conformational space further adding to the uncertainty [1,3]. In addition, the environment of the protein–the cytoplasm or the membrane–is represented in an implicit and static way, adding another layer of inaccuracy in free energy evaluation.

The *de novo* protein structure prediction algorithm BCL::Fold [6] was developed to overcome aforementioned problems and efficiently predict the topology of larger proteins with up to 400 residues. The necessary complexity reduction of the sampling space is achieved by assembling predicted secondary structure elements (SSEs) using a Monte Carlo algorithm and omitting more flexible loop regions. The energy evaluation of the sampled models is conducted using knowledge-based scoring functions [7], which provide a rapid way to approximate the free energy of the sampled conformation. In a previous study, it was demonstrated that BCL::Fold is able to efficiently sample the topologies of larger proteins [8]. Problems in model discrimination, which can arise from necessary simplifications made to sampling, scoring, and system representation, can be compensated for through incorporation of limited experimental data from electron microscopy [9–11], nuclear magnetic resonance spectroscopy [12], electron paramagnetic resonance spectroscopy [13,14], cross-linking experiments [15], small angle X-ray and neutron scattering [16], and predicted residue-residue contacts.

To evaluate the accuracy of our protein structure prediction pipeline, we participated in the community-wide *Critical Assessment of Protein Structure Prediction* (*CASP*) experiment in 2014 (*CASP11*), which takes place every two years [17]. Due to its setup as a double-blind study, the *CASP* experiment provides an impartial benchmark for protein structure prediction algorithms. The experimentally determined tertiary structures of the benchmark proteins are withheld from predictors, assessors, and organizers until conclusion of the experiment. After conclusion of the experiment, the experimentally determined structures are released to predictors and assessors and the predicted structures are released to the assessors, who determine the accuracy of the predictions. At the *CASP11* experiment, the amino acid sequences of fifty-five proteins were released to human predictors as regular targets (T0), i.e. without additional experimental restraints. Several regular targets were rereleased as 'assisted' targets with additional structural information in terms of predicted residue-residue contacts (TP), only correct residue-residue contacts (TC), nuclear magnetic resonance (NMR) nuclear overhauser effect (NOE) restraints (TS), and mass spectrometry (MS) cross-linking (XL) restraints (TX). As of June 2015, experimentally determined structures have been released for thirty regular protein targets. Of those, we predicted the tertiary structure of twenty targets during the *CASP11* experiment. Therefore, the analyses in this study are based on twenty T0, twelve TP, twelve TC, eight TS, and one TX protein target (Table 1).

**Table 1:** The proteins used in this study for the CASP11 benchmark.

Target	#res	#ɑ	#β	CO	PDB ID	Source	Resol. [Å]	Predicted as	Baker [GDT_TS]	Zhang [GDT_TS]	Kihara [GDT_TS]
T0759	113	5	6	24	4Q28	X-ray	2.6	-	38	40	32
T0761	252	5	13	55	4PW1	X-ray	2.1	TP, TS, TC	14	15	16
T0763	134	3	11	52	4Q0Y	X-ray	1.7	TP, TS, TC	15	19	18
T0765	98	3	4	59	4PWU	X-ray	2.5	-	74	79	47
T0767	296	8	15	57	4QPV	X-ray	1.8	TP, TS, TC, TX	13	16	15
T0769	112	2	4	44	2MQ8	NMR	4.3*	-	75	81	74
T0771	186	3	10	56	4QE0	X-ray	1.9	-	23	24	22
T0781	390	12	17	75	4QAN	X-ray	2.1	-	17	17	11
T0783	411	14	20	52	4CVH	X-ray	2.4	-	44	47	44
T0785	145	1	9	30	4D0V	X-ray	1.7	TP, TS, TC	26	27	26
T0794	506	6	28	61	4CYF	X-ray	2.3	TP, TS, TC	45	44	36
T0803	520	2	12	34	4OGM	X-ray	2.2	-	52	40	38
T0814	403	3	38	78	4R7F	X-ray	2.3	TP, TS, TC	11	15	15
T0818	138	4	12	34	4R1K	X-ray	2.6	TP, TS, TC	35	42	43
T0831	420	15	2	116	4QN1	X-ray	2.5	TP, TC	16	16	16
T0832	241	11	0	67	4RD8	X-ray	1.7	TP, TS, TC	13	17	17
T0834	222	10	6	51	4R7Q	X-ray	2.0	TP, TC	14	16	20
T0848	326	9	18	50	4R4G	X-ray	2.6	TP, TC	30	29	28
T0853	152	3	10	38	2MQB	NMR	1.0*	TP, TC	16	35	31
T0855	119	4	6	37	2MQD	NMR	1.3*	-	40	45	45

Twenty regular protein targets from the CASP11 benchmark set were used in this study. The proteins covered a wide range of structural features, like the sequence length (#res), the number of α-helices and β-strands (#α and #β), as well as the complexity of their fold quantified through the contact order metric (CO). Several regular targets were rereleased with limited experimental data in terms of predicted residue-residue contacts (TP). Only correct residue-residue contacts (TC), NMR-NOE restraints (TS), and MS-XL restraints (TX). The GDT_TS values of the submitted models are shown for three other groups (Baker [18], Zhang [19,20], and Kihara [21]) for comparison.

* Mean RMSD100 value between the models in the NMR ensemble

In the Materials and Methods section, we describe in detail the protein structure prediction pipeline employed in the *CASP11* experiment. In addition, we define the different quality metrics used in this study and we introduce the subset of the *CASP11* benchmark set analyzed in this study. The Results section reports the accuracy gained from the different pipeline modules and describes the protein structure prediction pipeline on hand for one regular target in detail. The Discussion section discusses the successes and failures of our pipeline.

## Materials and Methods

This section describes the different modules of the employed protein structure prediction pipeline, followed by a subsection describing how clustering was used to aggregate and transfer models between the different pipeline modules. Subsequent subsections describe the quality metrics used to quantify the results in terms of sampling accuracy and model discrimination. This section is concluded by a summary of the proteins used in this study.

### Low-Resolution Topology Search with BCL::Fold

BCL::Fold was specifically developed to predict the topologies of large proteins with a low-resolution approach. The BCL::Fold method was specifically designed to complement Rosetta by predicting SSE-only models with likely topologies of the protein and feeding them into Rosetta for loop and side chain construction as well as high-resolution refinement. The complexity of the conformational space grows exponentially with the number of residues in the protein, rendering exhaustive sampling of the conformational space impossible even for proteins with sequence lengths less than 100 residues. Protein structure prediction groups have come up with different approaches to address this problem. For example, Rosetta assembles the tertiary structure of proteins by assembling short fragments collected from the Protein Data Bank (PDB). This approach substantially reduces the complexity of the sampling space because the dihedral angles are not exhaustively sampled. Using rotamer libraries provides a similar simplification for the side chain conformations. However, even with mentioned simplifications the size of the conformational space remains too large for many proteins with more than 100 residues. Additionally, previous studies found that *de novo* prediction with Rosetta has a bias towards structures with low contact order [22].

Unlike Rosetta, BCL::Fold assembles disconnected fragments with limited internal flexibility to remove this bias. Secondary structure prediction methods are employed to assign the secondary structure to the sequence. For the resulting secondary structure elements (SSEs), initial conformations are created from idealized dihedral angles (φ, ψ): (-60°, -40°) for α-helices and (-135°, 135°) for β-strands. BCL::Fold assembles the SSEs in the three-dimensional space using a Monte Carlo Metropolis algorithm. Unlike in Rosetta, loop regions connecting the SSEs are not constructed explicitly, further reducing the complexity of the sampling and allowing for changing the overall topology in a small number of Monte Carlo steps. Instead the likelihood of the loop being able to close on the current conformation is predicted. Further complexity reduction is achieved by representing the side chains as “superatoms”, avoiding sampling of side chain conformations. BCL::Fold has this approach in common with Rosetta and other modeling approaches. Although these simplifications of the structural representation allow for an efficient enumeration of different topologies, a high-resolution scoring is no longer possible. BCL::Fold employs low-resolution scoring functions to evaluate geometrical parameters of the models created. These scoring functions include the likelihood of closing a loop given the number of amino acids and the Euclidean distance between two SSEs or if the twist angle between SSEs allows for side chain interaction among others. Most scoring functions used in BCL::Fold are knowledge-based, meaning they are derived from statistics over known protein structures deposited in the PDB and based on the inverse Boltzmann relation $E=-RT\times ln\frac{P_{obs}}{P_{back}}$, with *P*_*obs*_ being the probability of observing a specific feature and *P*_*back*_ being the probability of observing the specific feature by chance. The normalization by *P*_*back*_ is conducted to ensure that favorable states are assigned a negative score and unfavorable states are assigned a positive score. For example, the scoring function evaluating the burial of residues quantifies the degree of burial using the neighbor count metric [23]. For each amino acid type, the occurrences of its neighbor count values were collected from structures deposited in the PDB. The values were binned and the probability of each bin was computed and used as *P*_*obs*_. The background probability *P*_*back*_ was in this case the normalized sum of all normalized amino acid exposure distributions [7]. The BCL scoring function is the weighted sum of all scoring terms.

### Protein Structure Prediction Pipeline

**Fig 1:**
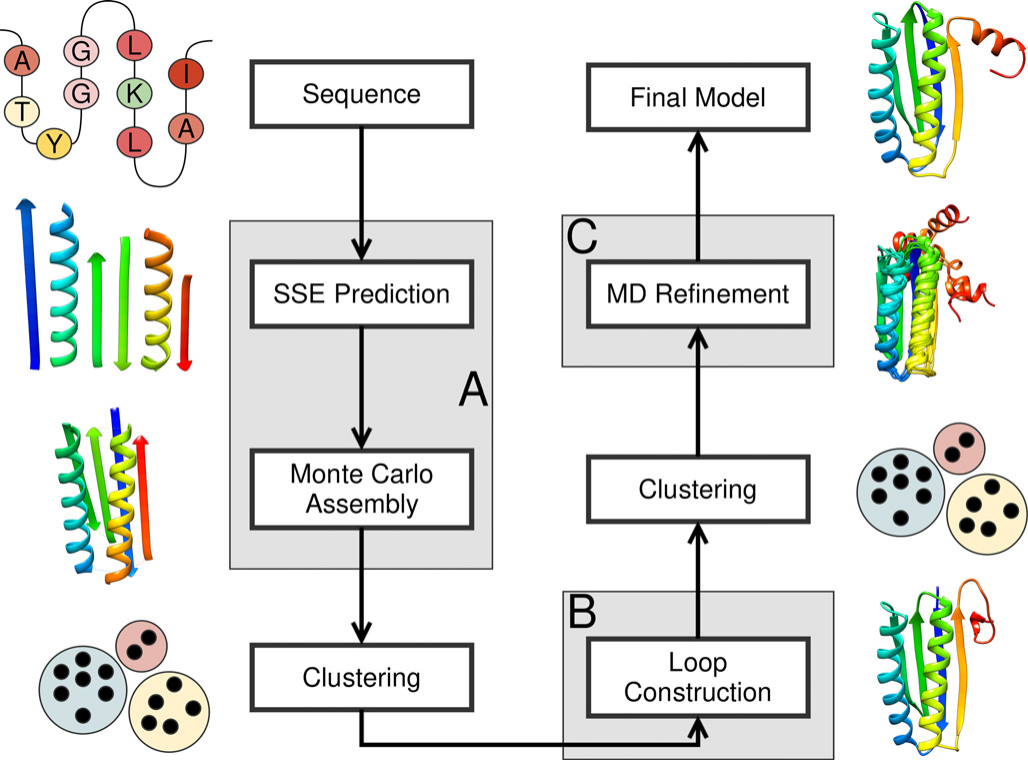
Protein structure prediction pipeline. The protein structure prediction pipeline employed in this study consisted of three modules–low-resolution topology search (A), high-resolution refinement and loop construction (B), and MD refinement (C). (A) The low-resolution topology search is based on BCL::Fold and uses machine learning algorithms to predict the secondary structure elements (SSEs) of the protein, which are subsequently arranged in the three-dimensional space using a Monte Carlo Metropolis algorithm. (B) High-resolution refinement and loop construction takes place using Rosetta’s cyclic coordinate descent algorithm followed by model relaxation. (C) Molecular dynamics simulations were conducted using the Amber package.

The protein structure prediction pipeline consisted of three modules (Fig 1). The first module consisted of a low-resolution topology search, which applied large-scale structural perturbations to the model in conjunction with a rapid low-resolution scoring function (Procedure A in S1 Protocol). The second module consisted of a high-resolution structural refinement, which only applied small-scale structural perturbations to the model in conjunction with a high-resolution scoring function while also constructing loop regions and placing the side chains (Procedure C in S1 Protocol). The third module consisted of a molecular dynamics (MD) simulation for further structural refinement and evaluation of model stability. The three modules were connected through filtering and clustering steps (Procedure B in S1 Protocol).

The protocol for the first module was based on the protein structure prediction protocol of BCL::Fold for soluble proteins [6]. In a first step, the secondary structure prediction methods Jufo9D [24], PsiPred [25], and MASP [26] were employed to predict the protein’s secondary structure. The protein’s tertiary structure was subsequently assembled from the predicted SSEs through a Monte Carlo sampling algorithm with a Metropolis criterion. After each Monte Carlo step, the model was evaluated using the weighted sum of multiple knowledge-based scoring functions including SSE packing, radius of gyration, residue exposure, residue pairing, loop closure geometry as well as residue-residue and SSE-SSE clashes [7]. Depending on the score difference to the previous Monte Carlo step and the simulated temperature, the new model was either accepted or rejected by the Metropolis criterion. The Monte Carlo Metropolis optimization was broken into six stages. The first five stages consisted of large-scale structural perturbations to search the energy landscape for minima. The employed perturbations included adding SSEs from the predicted SSE pool, removing SSEs from the model, large-scale translations and rotations of SSEs as well as the flipping and swapping of SSEs and SSE domains. Over the course of the first five stages, the weights of the scores evaluating clashes between residues and SSEs ramped up from 0 to 125, 250, 375, and 500. The five stages applying large-scale structural perturbations were followed by one stage of small-scale structural perturbations to transfer the model to the local energy minimum. If residue-residue contacts, nuclear overhauser effect (NOE) data, or cross-linking (MS-XL) data were available, the scoring function was extended by the appropriate scoring terms [12,15]. For each protein target, the first module sampled 20,000 SSE-only models without side chains or loop regions.

On conclusion of the first module, the models were ranked according to completeness. The 25% to 50% of the models with the lowest completeness were filtered out. The filtering threshold was chosen in dependence of the maximum completeness achieved throughout the conformation sampling. For the different targets, 10,000 to 15,000 models remained. For the remaining models, clustering was used to detect limit points in the sampling space, which indicate energy minima. The clustering was performed using a k-means implementation in the R package. For the different targets, this resulted in 10 to 50 clusters. The cluster medoids were subsequently selected as start models for the second module.

The protocol for the second module was based on Rosetta [27,28], added loop regions and side chains to the model, and conducted a high-resolution refinement. For each of the models resulting from the previous clustering step, 1,000 models were sampled using Rosetta’s cyclic coordinate descent algorithm [28] followed by model relaxation using Rosetta’s *‘relax’* application [27]. Per target, this module resulted in 10,000 to 50,000 models.

On conclusion of the second module, the models were ranked according to their Rosetta score and the 80% of the models with the worst score were discarded. The remaining 2,000 to 10,000 models were clustered according to the same criteria as the first clustering step. After filtering out clusters with a population of less than 0.5% of all models, this step resulted in 10 to 35 clusters. The cluster medoids were subsequently selected for high-resolution refinement and stability evaluation through MD simulations.

The third module consisted of MD simulations using the Amber package [29]. Hierarchical clustering was used to identify the sub-states for each model. Subsequently, a representative of each cluster was relaxed and scored using Rosetta. This module resulted in 10 to 35 models, which were visually inspected. From these models, five models were selected for submission based on their Rosetta scores.

### Using Clustering for Model Selection

The RMSD100 metric [30], which is the protein-size normalized root-mean-square deviation (RMSD) of the C_ɑ_-coordinates and computed as $RMSD100=RMSD/ln\left(\sqrt{\left(n/100\right)}\right)$, with *n* being the number of residues in the protein, was used to quantify the distance between models. The set of all models was sorted by their score and divided into the disjoint sets *high* and *low*. The set *low* contained the 20% of the models with the most favorable score, whereas the set *high* contained the remaining models. Both sets were clustered independently. The number of clusters was optimized to minimize the cluster radii and to maximize the separation between clusters, with an allowed maximum radius of 5 Å. Clusters that contained less than 0.5% of all models were also filtered out. The clustering after loop construction and side chain placement was conducted the same way as the clustering after the low-resolution homology search, but only the 20% of the models with the most favorable Rosetta score were considered.

### Molecular Dynamics Simulations

All simulations were prepared using Tleap [29] and simulations were performed with the Amber package [29] using the ffSB98ildn force field [31]. Each refinement target was solvated in a 10 Å TIP3P [32] water box with neutralizing Na^+^ or Cl^-^ ions then equilibrated following a modified procedure [33]. First, the solvent was minimized for 500 steps using steepest descent, followed by 5,000 steps of conjugate gradient minimization. Next, the systems were heated from 100 K to 300 K over 20ps with 500 kcal/mol/Å^2^ restraints on the protein followed by 30 ps of NPT at 300 K and 1 atm pressure. This process was repeated with restraints of 100, 50, 25, 12.5, and 1 kcal/mol/Å^2^. After equilibration, each structure consisted of a 50 ns NPT production run at 300 K with periodic boundary conditions using Langevin dynamics [34] with a collision frequency of 5 ps^-1^. The electrostatics were calculated using particle mesh-ewald [35] while a 10 Å cut-off was used to calculate long-range interactions The SHAKE [36,37] algorithm constrained all covalent bonds with hydrogen atoms allowing a 2 fs time step. Each production trajectory was analyzed using Cpptraj [38]. Hierarchical clustering using complete-linkage was used to identify all sub-states for each model. Subsequently, one representative from each cluster scored with the Rosetta [27] application.

### Evaluation of the Prediction Accuracy

Sampling accuracy and model discrimination were evaluated. The sampling accuracy was quantified using the *global distance test total score* (GDT_TS) [39] metric. The GDT_TS is the average percentage of C_ɑ_-coordinates in the model with a maximum deviation of 1 Å, 2 Å, 4 Å, and 8 Å from the experimentally determined structure. The GDT_TS is computed as *GDT*_*TS* = (*P*_1_+*P*_2_+*P*_4_+*P*_8_) / 4, with *P*_*i*_ being the percentage of residues in the model that can be superimposed with maximum deviation of *i* Å from the experimentally determined structure. Model discrimination is quantified through the enrichment metric, which equates to the percentage of the most accurate models that can be selected by the scoring function.

### Computation of Enrichments

The enrichment describes the correlation between model accuracy and score; thus, quantifying how well the scoring function is able to distinguish accurate models from inaccurate models. To compute the enrichment, the set of the sampled models *S* is divided into the disjoint subsets *P* (positive) and *N* (negative). The positive set contains the 10% of the models in *S*, which have the lowest RMSD100 value. The negative set contains the remaining models in *S*. In a second step, *S* is divided again into the disjoint subsets *PS* (positive score) and *NS* (negative score). The set *PS* contains the 10% of the models in *S*, which have the best score, whereas the set *NS* contains the remaining models. By computing the intersection *TP* = *P* ∩ *PS*, the set of the models, which can be identified by the scoring function, can be calculated. The enrichment is then calculated as *e* = |*TP*|/|*P*| ⋅ *10*, thus describing which fraction of the most accurate models can be identified by the scoring function. Therefore, the enrichment can span a range from 0 to 10, with 1 indicating random selection, enrichments larger than 1 indicating that the scoring function has the ability to recognize native-like models, and an enrichment of less than 1 indicating that the scoring function is selecting against accurate models.

### The CASP11 Benchmark Subset Used in This Study

The analyses in this study are based on twenty soluble proteins released as targets during the *CASP11* experiment. The twenty benchmark proteins covered a wide range of structural properties (Table 1), making them an appropriate test case for protein structure prediction algorithms. The sequence length ranged from 109 to 470 residues and the secondary structure content ranged from 6 to 41 SSEs. SSE definitions were obtained through DSSP [40]. The ɑ-helical content ranged from 1 to 15 SSEs, whereas the β-strand content ranged from 0 to 38 SSEs. The fold complexity quantified through the contact order metric [41] ranged from 34 to 116. Twelve of the twenty regular targets were also studied using with additional structural data such as residue-residue contacts, NMR-NOE restraints, or MS-XL restraints (Table 1).

### The Available Experimental Data

For twelve protein targets, limited experimental data was provided by the CASP organizers. The experimental data included predicted residue-residue contacts (TP and TC), NMR-NOE restraints (TS), and MS-XL restraints (TX). The residue-residue contacts were predicted by research groups participating in the CASP contact prediction experiment and included correct and incorrect residue-residue contacts for the TP targets. After completion of the TP predictions, a subset of the contacts only containing correct residue-residue contacts was released. The NMR-NOE restraints were simulated by Gaetano Montelione’s group and incorrect restraints were added purposefully. The XL-MS restraints were determined experimentally by Juri Rappsilber’s group.

## Results

This section is divided into subsections discussing the sampling accuracy and model discrimination of the low-resolution topology search module, followed by a subsection discussing the general decay of model accuracy over the course of the protein structure prediction pipeline. Subsequently, a case study for target T0769 describes in detail the processing of the data through the protein structure prediction pipeline. This section is concluded by a subsection describing the impact of different types of limited experimental data on protein structure prediction accuracy.

### BCL::Fold Sampled Models with a GDT_TS Value Greater than 30% for Twelve Out of Twenty Regular Targets

**Fig 2:**
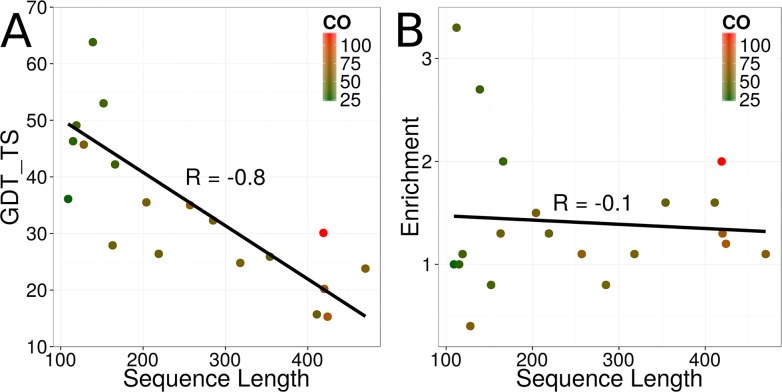
Sampling accuracy and model discrimination for the protein targets. (A) GDT_TS value of the most accurate model for each of the twenty regular targets sampled by the low-resolution topology search in dependence of the protein’s sequence length. The coloring is according to the complexity of the protein’s fold as quantified through the contact order metric (CO). (B) Model discrimination for each of the twenty regular targets as quantified through the enrichment metric in dependence of the protein’s sequence length. The coloring is according to the complexity of the protein’s fold quantified through the contact order metric (CO).

To quantify the ability of BCL::Fold to sample the topology of the target proteins, the GDT_TS metric was used. The GDT_TS computes the average percentage of C_ɑ_-coordinates in the model that deviate maximally 1 Å, 2 Å, 4 Å, and 8 Å from the experimentally determined structure (see Materials and Methods). For twelve out of twenty regular targets, BCL::Fold sampled models with a GDT_TS value greater than 30% (Table 2 and Fig 2A). The average GDT_TS value over all twenty regular targets was 36% (Table 2). The success in sampling accurate models strongly depended on the length of the protein's sequence (R-value of -0.8, Table 2 and Fig 2). Notably, there was no dependence on the complexity of the protein's topology as quantified through the contact order metric (R-value of 0.0).

**Table 2:** Model accuracy decay over the course of the pipeline.

	Fold		Cluster	Loop	Cluster	MD
Target	GDT_TS	e	GDT_TS	GDT_TS	GDT_TS	GDT_TS
T0759	36	1.0	29	26	21	22
T0761	32	0.8	29	19	15	17
T0763	28	1.3	20	24	19	19
T0765	46	0.4	32	50	26	26
T0767	25	1.1	20	15	13	13
T0769	74	3.3	66	69	66	77
T0771	36	1.5	27	22	18	18
T0781	20	1.3	15	12	9	9
T0783	16	1.6	10	13	10	9
T0785	46	1.0	40	26	22	22
T0794	24	1.1	14	11	10	8
T0803	64	2.7	13	26	21	16
T0814	15	1.2	10	9	7	8
T0818	42	2.0	37	34	24	18
T0831	30	2.0	21	17	14	12
T0832	35	1.1	25	22	19	19
T0834	26	1.3	19	15	12	10
T0848	26	1.6	20	12	11	11
T0853	53	0.8	42	27	23	17
T0855	49	1.1	40	33	28	18
∅	36	1.4	26	24	20	18

The quality of the most accurate models decayed over the course of the protein structure prediction pipeline. For each pipeline module, the GDT_TS value of the most accurate model is shown. For the low-resolution topology search, also the enrichment (e) is shown.

### The BCL::Fold Scoring Function Was Frequently Unable to Select Accurate Models

After conclusion of the first pipeline module–the low-resolution topology search–models were selected for high-resolution refinement and loop construction with Rosetta. Although the model selection was conducted using a clustering approach, how well the BCL::Fold scoring function identifies accurate models remains an interesting question. The ability of the scoring function to select the accurate models among the sampled models was quantified using the enrichment metric (see Materials and Methods), which computes the percentage of the most accurate models that can be selected by the scoring function.

Over all twenty regular targets the average enrichment was 1.4 (Table 2), meaning that 14% of the most accurate 10% models could be selected by the BCL::Fold scoring function, which is only slightly better than random selection. There was no clear correlation between the enrichment and the sequence length, the complexity of the protein’s fold, or the number of ɑ-helices and β-strands in the protein (S1 Fig). However, the model selection in our pipeline was not conducted through direct usage of the BCL score, but through clustering to identify limit points, which indicate score minima. To evaluate the success of this approach, we computed for each protein target the percentage of models that had a GDT_TS value greater or equals 40%, assuming with a high enough percentage, those models can be detected through clustering. A density in this context could be seen as significant if it surpassed the population cutoff of 0.5% during the first clustering step. For the regular targets T0769, T0785, T0803, T0853, and T0855 significant densities accounting for 54%, 1%, 47%, 4%, and 1% of all models could be detected. For the remaining targets, the percentages of models with a correct topology were below 0.5%. Notably, for four out of the five of the aforementioned protein targets, models with a GDT_TS value greater or equals 40% could be detected through clustering (Table 2).

### Model Accuracy Decayed over the Course of the Pipeline

**Fig 3:**
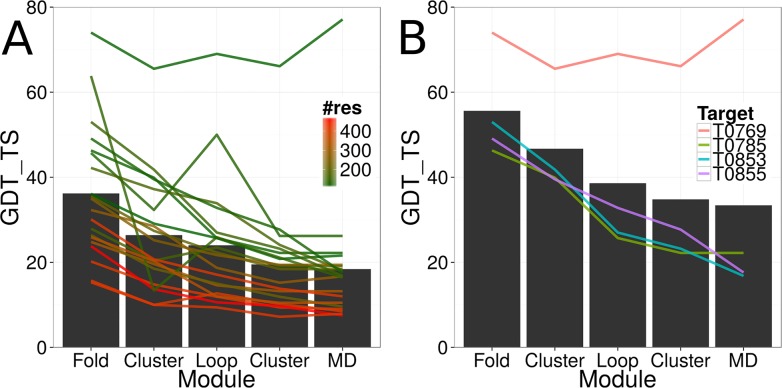
Model accuracy decay over the course of the protein structure prediction pipeline. (A) The model accuracy decayed over the course of the protein structure prediction pipeline. The black bars show the average GDT_TS value of the most accurate model over all twenty regular targets after each pipeline module. The lines show the development of model accuracy for each target over the course of the pipeline. The coloring is according to the number of residues in the protein target. (B) Same as in (A) for four selected targets with a GDT_TS value of greater than 40% after the first clustering step.

The three different modules of our protein structure prediction pipeline were connected through filtering and clustering. In an optimal scenario, the most accurate models would be detected through clustering and transferred to the subsequent module. However, ambiguities in the employed scoring function and the consequently biased sampling lead to difficulties in detecting the most accurate models. In clustering, native-like conformations become detectable if a sufficiently high density of models exists around it. For the four targets T0769, T0785, T0853, and T0855, models with a GDT_TS value greater or equals 40% could be detected through clustering after the low-resolution topology search and transferred to the second module for loop construction and side chain placement (Table 2 and Fig 3B). The average GDT_TS value of the most accurate models for the four regular targets developed from 56% to 47%, and to 39% over the course of the low-resolution topology search, the first clustering, and the loop construction and side chain placement steps (Fig 3B). At general, a decay of model accuracy was observable over the course of the protein structure prediction pipeline (Table 2 and Fig 3A). The average GDT_TS values over all twenty regular targets dropped from 36% (low-resolution topology search) to 26% (first clustering), to 24% (loop construction and side chain placement), to 20% (second clustering), and to 18% (MD refinement). Expectedly, the most significant loss in model accuracy happened during the transition for the low-resolution topology search to loop construction and side chain placement where the average GDT_TS value over all twenty regular targets dropped from 36% to 24%. A significant improvement through MD refinement could only be observed for regular target T0769 for which the GDT_TS value of the most accurate model improved from 66% to 77%. For the other regular targets, the GDT_TS value of the most accurate start model was 27% or less and MD refinement consequently was not able to improve the accuracy of the model. For the previously mentioned regular target T0765, the most accurate models sampled by the loop construction and side chain placement module could not be detected through the clustering and filtering step before MD refinement.

### A Case Study of Regular Target T0769

**Fig 4:**
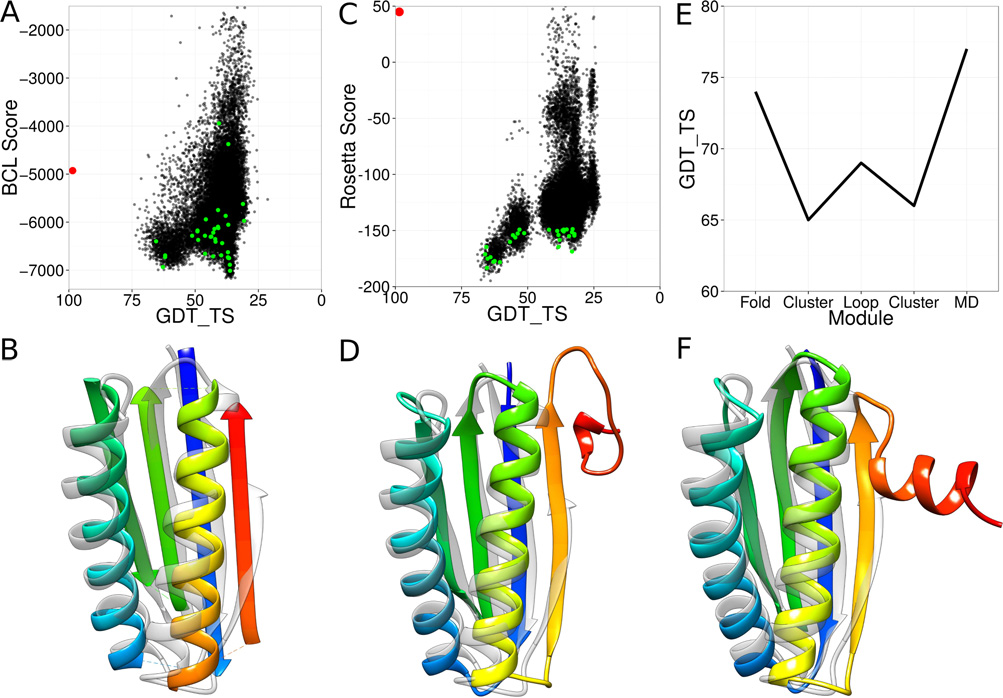
Case study of regular target T0769. (A) Results for the low-resolution topology search. Each black dot represents one sampled model. The NMR structure is shown in red. The green dots are the cluster medoids selected after the topology search. (B) Most accurate model after the topology search (rainbow) superimposed with the NMR structure (grey). (C) Results for high-resolution refinement and loop construction. Each black dot stands for one sampled model. The NMR structure is shown in red. The green dots are the cluster medoids selected after the high-resolution refinement. (D) Most accurate model after the high-resolution refinement (rainbow) superimposed with the NMR structure (grey). (E) Development of the GDT_TS of the most accurate model over the course of the pipeline. (F) Most accurate model after the molecular dynamics refinement (rainbow) superimposed with the NMR structure (grey).

The regular target T0769 was a 112-residue-long soluble protein consisting of two ɑ-helices and four β-strands, resembling a ferredoxin fold. The first module of our protein structure prediction pipeline—the low-resolution topology search—sampled models with GDT_TS values of up to 74% (Table 2 and Fig 4A). An enrichment of 3.3 was observed indicating that 33% of the 10% most accurate models could be selected by the scoring function. About 69% of all models had the correct topology. Through clustering, a model with a GDT_TS value of 65% could be detected (Table 2 and Fig 4B). In the second module of the pipeline, the loop regions were constructed and the side chains were placed. The most accurate model resulting from this pipeline module arrived at a GDT_TS value of 69% (Fig 4C and 4D). The models resulting from the second module were clustered again and the cluster medoids selected for MD refinement. The most accurate medoid had a GDT_TS value of 66%. Upon conclusion of the MD simulations, the refined models were rescored using Rosetta and the model with the most favorable Rosetta score was designated as final model. The final model arrived at a GDT_TS value of 77% (Fig 4E and 4F).

### The Impact of Limited Experimental Data on Protein Structure Prediction Accuracy

If none of the participating groups in the *CASP11* experiment was able to accurately predict the tertiary structure of a regular target, this target was rereleased as 'assisted' target and additional limited experimental data was provided. Of the twenty regular targets analyzed in this study, twelve targets were rereleased as ‘assisted’ targets (Table 1). Of those, predicted residue-residue contacts (TP) and only correct residue-residue contacts (TC) were provided for all twelve assisted targets. NMR-NOE data (TS) was provided for eight assisted targets, and MS-XL data was provided for one assisted target (TX). To evaluate the impact of different kinds of experimental data on the sampling accuracy of the low-resolution topology search module, we compared the average GDT_TS value of the ten most accurate models (μ_10_) for each restraint type and protein target. The comparison is based on ten models instead of one model to account for the randomness of the sampling. The impact of limited experimental data on model discrimination was evaluated by comparing the achieved enrichments (see Materials and Methods).

**Fig 5:**
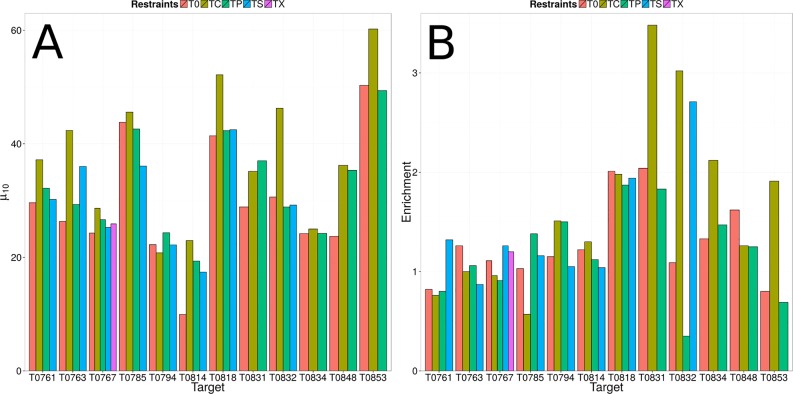
Sampling accuracy and model discrimination for 'assisted' targets. (A,B) The average GDT_TS values of the most accurate models (μ_10_) and the enrichments are compared for protein structure prediction without restraints (T0), with predicted residue-residue contacts (TP), only correct residue-residue contacts (TC), NMR-NOE restraints (TS), and MS-XL restraints (TX).

For the predicted residue-residue contacts (TP), which also include incorrect residue-residue contacts, only minor improvements in sampling accuracy could be observed. Whereas the average μ_10_ value over the twelve TP targets was 30% when predicting without residue-residue contacts, incorporating residue-residue contacts improved the average μ_10_ value to 33% (Table 3 and Fig 5A). There was also no beneficial impact on model discrimination. Actually, the average enrichment value dropped from 1.3 to 1.2 when using predicted residue-residue contacts. Incorporation of only correct residue-residue contacts (TC), had a more significant impact on the sampling accuracy, which is demonstrated by an improved average μ_10_ value of 38%. A similar beneficial impact could be observed on model discrimination, which is demonstrated by an improved enrichment value of 1.7 (Table 3 and Fig 5B). NMR-NOE restraints (TS) were only available for eight protein targets. For those eight protein targets, only minor improvements in sampling accuracy and model discrimination could be observed. The average μ_10_ and enrichment values improved from 29% to 30% and from 1.2 to 1.4, when compared to the prediction results without using additional structural information (Table 3 and Fig 5). MS-XL (TX) was only available for one regular target (T0767) analyzed in this study. For this protein target, incorporation of MS-XL data also only had a minor impact on the sampling accuracy and model discrimination. The μ_10_ and enrichment values improved from 24% to 26% and from 1.1 to 1.2 (Table 3 and Fig 5).

**Table 3:** Protein structure prediction results from limited experimental data.

	T0		TP		TC		TS		TX	
Target	μ_10_	e	μ_10_	e	μ_10_	e	μ_10_	e	μ_10_	e
T0761	30	0.8	32	0.8	37	0.8	30	1.3	-	-
T0763	26	1.3	29	1.1	42	1.0	36	0.9	-	-
T0767	24	1.1	27	0.9	29	1.0	25	1.3	26	1.2
T0785	44	1.0	43	1.4	46	0.6	36	1.2	-	-
T0794	22	1.1	24	1.5	21	1.5	22	1.1	-	-
T0814	10	1.2	19	1.1	23	1.3	17	1.0	-	-
T0818	41	2.0	42	1.9	52	2.0	43	1.9	-	-
T0831	29	2.0	37	1.8	35	3.5	-	-	-	-
T0832	31	1.1	29	0.4	46	3.0	29	2.7	-	-
T0834	24	1.3	24	1.5	25	2.1	-	-	-	-
T0848	24	1.6	35	1.2	36	1.3	-	-	-	-
T0853	50	0.8	49	0.7	60	1.9	-	-	-	-
∅	30	1.3	33	1.2	38	1.7	30	1.4	26	1.2

The average GDT_TS values of the ten most accurate models (μ_10_) and the enrichment (e) are shown for prediction from the primary structure alone (T0), from predicted residue-residue contacts (TP), only correct residue-residue contacts (TC), NMR-NOE restraints (TS), and MS-XL restraints (TX).

### The Low-Resolution Topology Search Fails in Some Instances to Sample the Correct Topology

**Fig 6:**
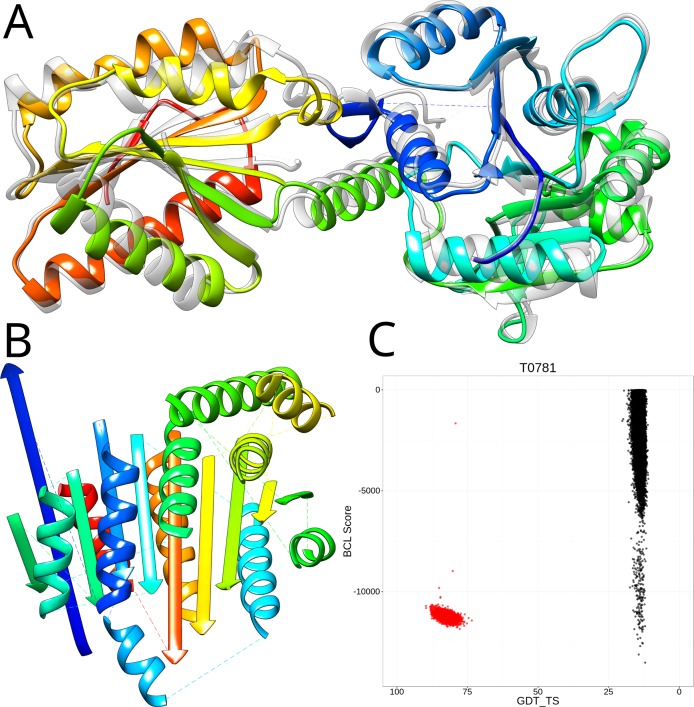
Limitations in the conformational sampling hinder structure prediction for regular target T0781. (A) Experimentally determined structure of T0781 (PDB entry 4QAN, grey) superimposed with the same structure after relaxation with the BCL scoring function (rainbow). (B) Best scoring de novo model predicted by BCL::Fold. (C) Shown are the BCL score of the models (y-axis) and the GDT_TS of the model relative to the experimentally determined structure (x-axis). Relaxing the experimentally determined structures in the BCL::Fold scoring function reveals native-like conformations with a favorable score (red dots). In comparison, the de novo folded conformations observed during the CASP experiment (black dots) achieve comparable scores but don’t include conformations, which are structurally similar to the experimentally determined structure.

In a Monte Carlo Metropolis algorithm, the sampling depends on the scoring because the probability with which a Monte Carlo step is accepted depends on the score difference to the previous Monte Carlo step [6]. To further investigate limitations in sampling and scoring, we relaxed the experimentally determined structures in the BCL::Fold force field. In this process, small structural perturbations are applied to the experimentally determined structure in order to find a structurally similar conformation with a more favorable BCL score. For sixteen out of the twenty benchmark proteins (80% of all targets), the relaxation resulted in structurally similar conformations (GDT_TS > 70%), which had a favorable BCL score (among the top 20% of the sampled models). We conclude that these topologies should therefore be selectable through the BCL scoring function and within the sampling range of BCL::Fold (Fig 6 and S2 Fig). For T0781, conformations with a GDT_TS greater than 80% exist (Fig 6C) that score as well as our best scoring *de novo* sampled conformations during the *CASP11* experiment (Fig 6). To further investigate, why none of the well scoring conformations were sampled, we folded an additional 500,000 conformations for T0781 with additional correct residue-residue contact restraints to further limit the size of the sampling space. Despite that, it was not possible to sample a conformation with a GDT_TS greater than 25%, which indicates that the sampling algorithm needs to be revisited. Visual inspection of a clustered representation of the sampled models revealed that the SSEs in all cluster medoids exhibited a strong bias towards Rossmann-like [42,43] α-β-α-sandwich topologies (Fig 6B), whereas the experimentally determined structure (PDB entry 4QAN) is categorized as α-β-roll (Fig 6A), according to a CATH [44] search. In a future step, the sampling of β-strand containing topologies needs to be thoroughly revisited.

For four benchmark targets (T0759, T0771, T0818, and T0831, see S2 Fig), the relaxation of the experimentally determined structure did not result in conformations with a score as favorable as the score of the *de novo* folded models. Whereas this did not pose any problem for target T0818 because conformations with favorable score and GDT_TS value > 40% exist (S2 Fig), this could have had detrimental effect on the structure prediction for the other three targets. The remaining targets are outliers to the statistics the BCL::Fold scoring function is based on (see Materials and Methods for detail). The scores of the targets T0759 and T0831 (PDB entries 4Q28 and 4QN1) are heavily penalized for their large radius of gyration–the spatial extent of the proteins’ tertiary structures with respect to their sequence lengths [7]. The radius of gyration score introduces a bias towards globular folds and it will have to be evaluated on a large benchmark set if turning off this scoring term will have a negative impact on structure prediction at general. For the remaining target T0771 (PDB entry 4QE0), multiple properties of the experimentally determined structure–burial of residues, residue-residue interactions, SSE packing–scored worse than the *de novo* predicted models and the scoring function was not able to identify a native-like conformation. This target represents an outlier to our statistics over protein structure properties and would have to be complemented with experimental restraints.

## Discussion

### Necessary Simplifications in the Topology Search Hinder Protein Structure Prediction

The vast size of the conformational space does not allow for exhaustive sampling of all possible conformations of a protein's chain. BCL::Fold reduces the complexity of the search space by assembling the protein's tertiary structure from idealized SSEs and only allowing for limited deviations from the idealized dihedral angles. Although this approach reportedly worked well for ɑ-helical proteins^5^ and, in particular, membrane proteins [45], the protein targets in the *CASP11* benchmark set contained many proteins with a large percentage of β-strand content (Table 1). Many of those proteins contained strongly bent β-strands, making it impossible for the low-resolution topology search module to sample and select models having the correct topology (Table 2 and Fig 2). Although BCL::Fold was able to sample models with a GDT_TS value of at least 40% for seven out of twenty regular targets, only four of those targets had accurate models in a sufficient density to be detectable through clustering (Table 2 and Fig 3). Consequently, future work needs to be focused on the development of efficient algorithms to assemble the topologies of β-sheet domains and domains significantly deviating from idealized dihedral angles at general.

### The High-Resolution Refinement Protocol Requires Additional Optimization

Over the course of the protein structure prediction pipeline, a general decay of model accuracy was observed (Table 2 and Fig 3). During the loop construction and side chain placement step using Rosetta, the average GDT_TS value of the most accurate models over all twenty regular targets dropped from 27% to 24% (Table 2 and Fig 3). Only for one regular target (T0765), a significant improvement in model accuracy could be observed. Those findings are less surprising since the Rosetta loop construction and refinement step, only applies small-scale perturbations to the start model, and therefore did not further explore the conformational space to transform a topologically incorrect model into an accurate conformation. Consequently, future work needs to be focused on the development of more accurate scoring functions to increase the sampling density of accurate models. A similar observation was made for the atomic-detail MD refinement step. The average GDT_TS value of the most accurate models over all twenty regular targets dropped from 20% to 18%. A significant improvement in model accuracy was only observed for one regular target (T0769), for which the GDT_TS value of the most accurate model improved from 66% to 77% (Table 2, Fig 3 and Fig 4). However, we cannot necessarily conclude that MD refinement is unable to recover from inaccurate starting models. Previous work by the groups of David E. Shaw, Chaok Seok, and J. Andrew McCammon demonstrated that MD refinement is able to improve the accuracy of a model [46–50]. An evaluation of the CASP11 refinements through MD also reports some success [51]. Whereas Shaw describes a successful approach using simulations at least 100 μs long, we employed 50 ns simulations. In conjunction with the low accuracy of our start models, this could explain why our MD refinement was in most cases unable to significantly improve the accuracy of the model. In upcoming studies, we will therefore employ longer simulations to allow for sufficient coverage of the conformational space. Additional influence factors originate in the employed force field, which will have to be investigated in future studies.

### Sampling Problems Could Not Be Overcome through Limited Experimental Data

Incorporation correct residue-residue contacts (TC) into the scoring function improved the average μ_10_ values for the twelve 'assisted' targets from 32% to 40% (Table 3 and Fig 5A). Statistically significant improvements in sampling accuracy were only observed for the six targets T0763, T0814, T0818, T0832, T0848, and T0853, for which an average improvement of 13% was observed. For the remaining targets, only minor improvements in sampling accuracy were observed, indicating that a conformation with high structural similarity to the experimentally determined structure is not part of the sampling space. The remaining twelve targets, for which no significant improvement could be observed, were either large or had contained a large number of β-strands. Expectedly, improvements in sampling accuracy and model discrimination by using NMR-NOE restraints and predicted residue-residue contact restraints were less pronounced, because those restraint sets also contained incorrect distance restraints. The NMR-NOE restraints were simulated and incorrect restraints were added purposefully by the CASP organizers (see Materials and Methods). Exemplary are the targets T0818 and T0832 for which incorporation of correct residue-residue contacts resulted in an improvement of the μ_10_ values from 41% and 31% to 52% and 46%, whereas incorporation of NMR-NOE and predicted residue-residue contact restraints did not result in any improvement (Table 3 and Fig 5A). Consequently, future work needs to be also focused on developing methods to properly handle incorrect experimental data.

## Supporting Information

S1 Fig No dependence of the enrichment on secondary structure content or contact order. No correlation between the enrichment and the percentage of α-helices (A), β-strands (B), or contact order (C) could be observed. In each case, the absolute value of the R-value was less than 0.1.
(PDF)

S2 Fig Score-accuracy correlations of de novo folded models and relaxed experimentally determined structures. Shown are the BCL score of the models (y-axis) and the GDT_TS of the models relative to the experimentally determined structure (x-axis). De novo folded models are depicted as black dots and models sampled through relaxation of the experimentally determined structure are shows as red dots.
(PDF)

S1 Protocol Protein structure prediction protocol. The following protocol requires an installation of the BioChemical Library (BCL), Rosetta, and R with the cluster package. The BCL license can be obtained at www.meilerlab.org/bclcommons. The Rosetta license can be obtained at www.rosettacommons.org.
(PDF)
