# Supplementary figures and images for: CASP11 – An Evaluation of a Modular BCL::Fold-Based Protein Structure Prediction Pipeline
Source: PLoS One. 2016 Apr 5;11(4):e0152517. doi: 10.1371/journal.pone.0152517 (PMC4821492; doi:10.1371/journal.pone.0152517)

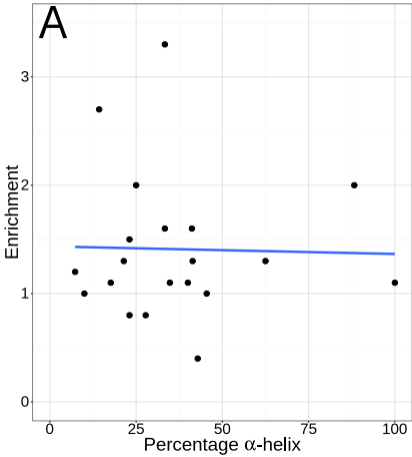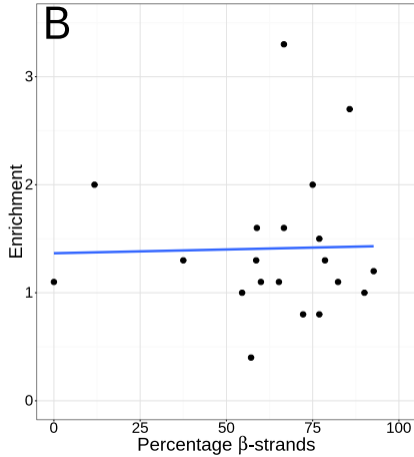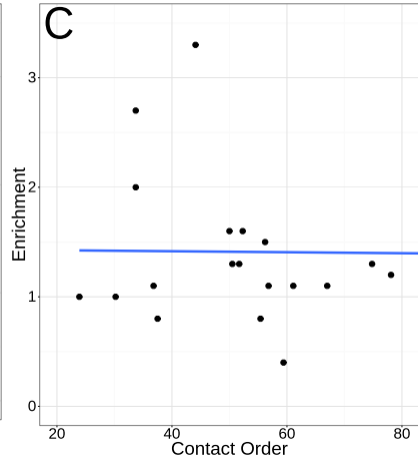

Supplement: S1 Fig — No correlation between the enrichment and the percentage of α-helices (A), β-strands (B), or contact order (C) could be observed. In each case, the absolute value of the R-value was less than 0.1. (PDF) [file pone.0152517.s001.pdf]

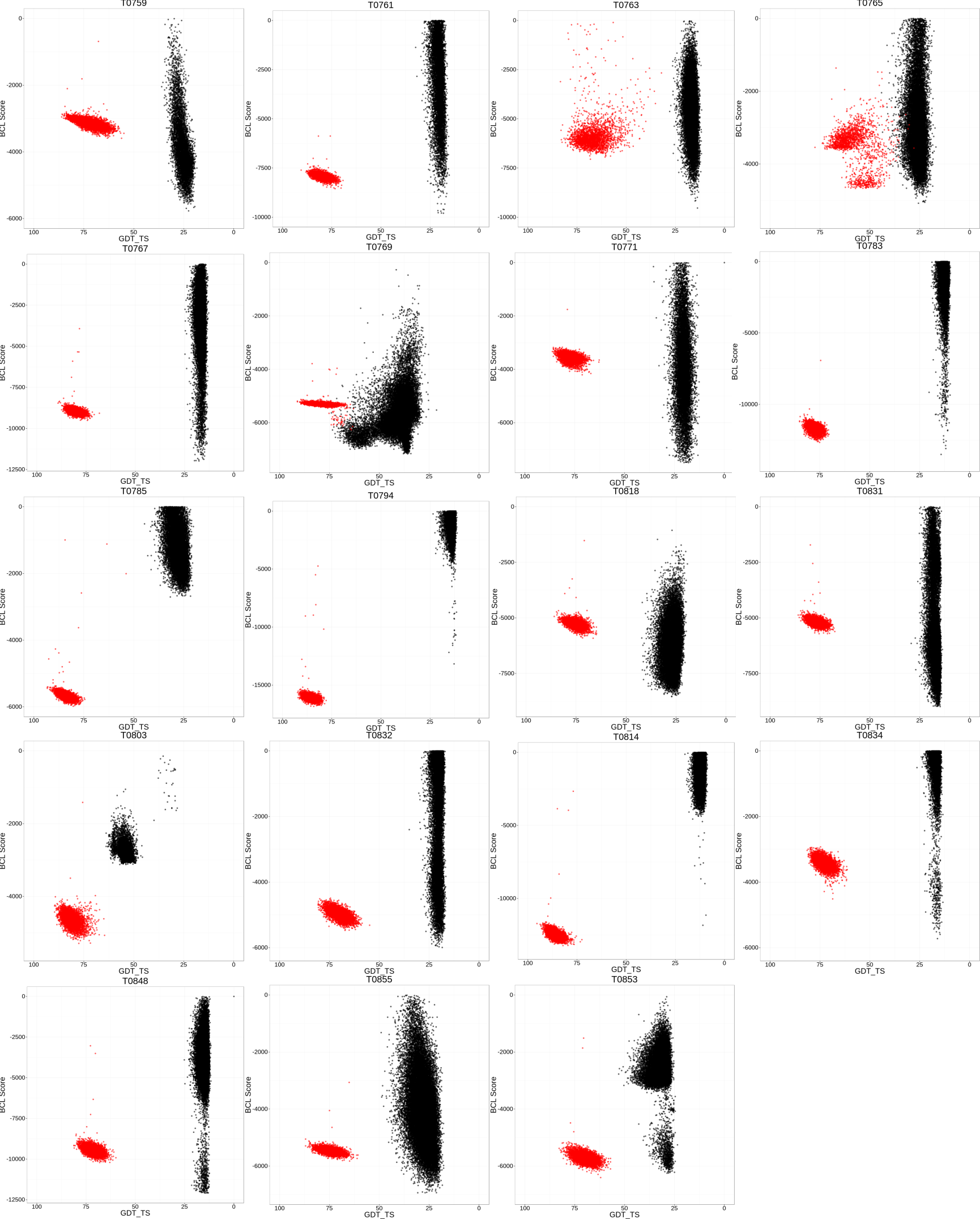

Supplement: S2 Fig — Shown are the BCL score of the models (y-axis) and the GDT_TS of the models relative to the experimentally determined structure (x-axis). De novo folded models are depicted as black dots and models sampled through relaxation of the experimentally determined structure are shows as red dots. (PDF) [file pone.0152517.s002.pdf]
