# Supplementary material for: CASP11 – An Evaluation of a Modular BCL::Fold-Based Protein Structure Prediction Pipeline
Source: PLoS One. 2016 Apr 5;11(4):e0152517. doi: 10.1371/journal.pone.0152517 (PMC4821492; doi:10.1371/journal.pone.0152517)
Supplement: S1 Protocol — The following protocol requires an installation of the BioChemical Library (BCL), Rosetta, and R with the cluster package. The BCL license can be obtained at www.meilerlab.org/bclcommons. The Rosetta license can be obtained at www.rosettacommons.org. (PDF) [file pone.0152517.s003.pdf]

Protein structure prediction protocol. The following protocol requires an installation of the BioChemical Library (BCL), Rosetta, and R with the cluster package. The BCL license can be obtained at www.meilerlab.org/bclcommons. The Rosetta license can be obtained at www.rosettacommons.org.

Procedure A: Low-resolution topology search

The low-resolution topology search was performed using BCL::Fold. The secondary structure element (SSE) prediction were performed using PsiPred, Jufo9D, and MASP. As a first step, an SSE pool is created from the SSE predictions. The directory <seq_dir> has to contain the SSE prediction files generated by PsiPred, Jufo9d, and MASP.

```
bcl.exe CreateSSEPool -prefix <seq_dir> -pool_min_sse_length 5 3 -ssmethods
JUFO9D PSIPRED MASP -sse_threshold 0.4 0.4 0.4 -factory SSPredThreshold
```

The SSEs in the SSE pool are subsequently arranged in the three-dimensional space using BCL::Fold. The command line below generate twenty models.

```
bcl.exe Fold -fasta <protein>.fasta -sequence_data <seq_dir> <protein> -
sspred JUFO9D PSIPRED -pool <protein>.pool -pool_separate -stages_read
stages.txt -protein_storage <output_dir> -nmodels 20 -opencl Disable
```

Procedure B: Clustering for model selection

The clustering was performed using the R package with cluster. In a first step, the pairwise RMSD100 distances between the sampled models has to be computed. The file *pdbs.ls* contains the file paths:

```
bcl.exe PDBCompare -pdb_list pdbs.ls -quality RMSD -norm100
```

The command line above results in a distance matrix with each row and column corresponding to one model. The models from the low-resolution topology search are subsequently clustered based on their pairwise distances using R:

```
// load the dissimilarity matrix created with the BCL
data_mat = as.matrix(read.table("distance_matrix.tbl", header = T))

// create a full matrix
data_mat = data_mat + t(data_mat)

// convert into a dissimilarity matrix
data_mat = as.dist(data_mat)

// cluster for k cluster centers
clusters = pam(data_mat, k)

// print out clustering information like diameter and separation
clusters$clusinfo

// print out cluster centers
clusters$medoids
```

Procedure C: Addition of loop and side chain coordinates

Loop regions and side chains as well as the high resolution refinement were performed using Rosetta. A prerequisite is the creation of the fragment files, which can be obtained from the Robetta server at http://robetta.bakerlab.org. Subsequently the loop regions are constructed. The command line below will sample ten models:

```
loopmodel @<protein>.options -database database/ -in:file:fasta
<protein>.fasta -in:file:s <protein>.pdb -loops:loop_file <protein>.loops -
out:prefix <output_path> -nstruct 10
```

The options file used is given below. 9.bin and 3.bin are the fragment files for the respective fragment lengths.

```
-loops:fa_input
-loops:frag_sizes 9 3 1
-loops:frag_files 9.bin 3.bin none
-loops:build_initial true
-loops:remodel quick_ccd
-loops:refine refine_ccd
-loops:extended true
-loops:relax relax
-ex1
-ex2
-out:output true
-out:pdb true
```
